# Considering the case for an antidepressant drug trial involving temporary deception: a qualitative enquiry of potential participants

**DOI:** 10.1186/1472-6963-7-64

**Published:** 2007-04-30

**Authors:** Christopher F Dowrick, John G Hughes, Julia J Hiscock, Mark Wigglesworth, Thomas J Walley

**Affiliations:** 1 Division of Primary Care, University of Liverpool, Whelan Building, Liverpool L69 3GB, UK; 2 National Primary Care Research and Development Centre, 5^th ^Floor, Williamson Building, University of Manchester, Oxford Road, M13 9PL, UK; 3 Aintree Park Group Practice, 46 Moss Lane, Orrell Park, Liverpool, L9 8AL, UK; 4 Prescribing Research Group, Division of Biomedical Sciences, Pharmacology and Therapeutics, University of Liverpool, L69 3GB, UK

## Abstract

**Background:**

Systematic reviews of randomised placebo controlled trials of antidepressant medication show small and decreasing differences between pharmacological and placebo arms. In part this finding may relate to methodological problems with conventional trial designs, including their assumption of additivity between drug and placebo trial arms. Balanced placebo designs, which include elements of deception, may address the additivity question, but pose substantial ethical and pragmatic problems. This study aimed to ascertain views of potential study participants of the ethics and pragmatics of various balanced placebo designs, in order to inform the design of future antidepressant drug trials.

**Methods:**

A qualitative approach was employed to explore the perspectives of general practitioners, psychiatrists, and patients with experience of depression. The doctors were chosen via purposive sampling, while patients were recruited through participating general practitioners. Three focus groups and 12 in-depth interviews were conducted. A vignette-based topic guide invited views on three deceptive strategies: post hoc, authorised and minimised deception. The focus groups and interviews were tape-recorded and transcribed. Transcripts were analysed thematically using Framework.

**Results:**

Deception in non-research situations was typically perceived as acceptable within specific parameters. All participants could see the potential utility of introducing deception into trial designs, however views on the acceptability of deception within antidepressant drug trials varied substantially. Authorized deception was the most commonly accepted strategy, though some thought this would reduce the effectiveness of the design because participants would correctly guess the deceptive element. The major issues that affected views about the acceptability of deception studies were the welfare and capacity of patients, practicalities of trial design, and the question of trust.

**Conclusion:**

There is a trade-off between pragmatic and ethical responses to the question of whether, and under what circumstances, elements of deception could be introduced into antidepressant drug trials. Ensuring adequate ethical safeguards within balanced placebo designs is likely to diminish their ability to address the crucial issue of additivity. The balanced placebo designs considered in this study are unlikely to be feasible in future trials of antidepressant medication. However there remains an urgent need to improve the quality of antidepressant drug trials.

## Background

### Is antidepressant medication useful?

There is considerable debate concerning the utility of antidepressant medication in the treatment of depressive disorders. The prevailing view amongst psychiatrists is that antidepressant medication is of proven efficacy in reducing the severity and duration of major depression and should therefore be used as its first line of treatment [1]. However systematic reviews of randomised double blind placebo controlled trials of antidepressant medication indicate that differences in outcome between drug and placebo arms are often marginal, and may be exaggerated by selective publication or even deliberate misrepresentation [2]. Consensus and editorial statements indicate an awareness that this raises important and unresolved questions [3,4].

In a review of 75 placebo controlled trials of antidepressant medication prescribed in outpatient settings, Walsh *et al *concluded that the placebo response is 'variable, substantial and growing'. They calculated that about 30 per cent of the patients who were assigned to placebo groups in these trials showed clinically significant improvement, with a range from 12.5 per cent to over 50 per cent. This compared with an average response of 50 per cent to the main active medication being tested in the trial [5].

Walsh *et al *report that the proportion of people responding to placebo, and also to active medication, has increased at a rate of about seven per cent per decade. As treatments for depression have become more widely available and socially acceptable, it has become easier to recruit members of the general public to take part in clinical trials, rather than relying on patients referred from other clinicians. As a result, it is possible that clinically important characteristics of patients taking part in treatment studies may have altered. For example people coming forward from the general public may have less chronic types of depression, or experience fewer contributory life difficulties, than those recruited through hospital clinics. It is also possible that the increased social acceptability of depression and its management have affected the beliefs and expectation of both clinicians and patients taking part in trials, which in turn may enhance placebo effects.

Kirsch *et al *analysed the antidepressant medication data submitted to the United States' drug regulatory agency, the Food and Drug Administration (FDA) [6]. This is a different database from that used by Walsh et al, which was based on peer reviewed published trials. Here they examined the studies presented as evidence by pharmaceutical companies to the United States government, on the basis of which licences were granted to market particular antidepressant drugs. Using Hamilton's depression rating scale as their benchmark, they found that the mean differences between responses to antidepressant drugs and placebo was only two points, within a range from 0 to 52 points. While this difference was statistically significant, its relevance in terms of clinical practice is questionable.

Khan *et al *also used the FDA database to evaluate clinical trial data from the nine antidepressants approved by the FDA between 1985 and 2000 [7]. These trials comprised 10,030 depressed patients who participated in 52 clinical trials evaluating 93 treatment arms. Less than half (48%, 45/93) of the antidepressant treatment arms showed superiority to placebo.

In combination, these reports raise important methodological issues:

First, the average length of antidepressant drug trials has increased significantly during the past twenty years, though few continue beyond 12 weeks. This gives more time for the cumulative effects of "best medical care" and non-specific interventions which are inherent and inevitable in clinical trials, and – importantly – provides a longer period during which spontaneous recovery could be observed [5].

Second, many drug trials now include a placebo 'run-in period', which is designed to exclude those patients with disorders which resolve spontaneously. This tends to increase the apparent efficacy of antidepressant medication.

Third, there is debate over the use of active (i.e. designed to mimic expected side effects of trial drug) or inert placebos, and the effect of this on the ability of doctors and patients to guess which trial arm they are in. Correctly guessing may have the effect of increasing observed differences between active and control arms [8], particularly in milder disorders where patients' beliefs about the efficacy or otherwise of medication affect their response to it [9].

Finally, we have the fundamental problem that clinical trials are based on the assumption of *additivity*. That is, the drug is deemed effective only if the response to it is significantly greater than the response to placebo, and the magnitude of the drug effect is assumed to be the difference between the response to drug and the placebo. However, this assumption is problematic [10].

### The problem of additivity

Clinical trials of antidepressant medication are based on the method of residues: the assumption that the magnitude of the drug effect is the difference between response to drug and placebo. However this assumption is simplistic. The placebo effect is not simply additive, but can be synergistic or even antagonistic, with a complex web of intricate variables influencing the healing response [11].

Alcohol and stimulant drugs, for example, produce at least some drug and placebo effects that are not additive. Placebo alcohol produces effects that are not observed when alcohol is administered surreptitiously, and alcohol produces effects that are not duplicated by placebo alcohol. The placebo and pharmacological effects of caffeine are additive for feelings of alertness but not for feelings of tension, and similarly mixed results have been reported for other stimulants [12].

If antidepressant drug effects and antidepressant placebo effects are not additive, the ameliorating effects of antidepressants might be obtained even if patients did not know the drug was being administered. If that is the case, then antidepressant drugs have substantial pharmacologic effects that are duplicated or masked by placebo. In this case, conventional clinical trials are inappropriate for testing the effects of these drugs, as they may result in the rejection of effective medications. Conversely, if drug and placebo effects of antidepressant medication are additive, then the data produced by Kirsch and others indicate that those effects are small, at best, and of questionable clinical efficacy. Finally, it is conceivable that the effects are partially additive, with the true drug effect being somewhere in between these extremes.

We do not know which of these models is most accurate, because the assumption of additivity has never been tested with antidepressant medication.

### Balanced placebo designs

One method of testing the additivity issue is the use of the *balanced placebo *design [10]. In this design participants are recruited for a study in which active drug or placebo will be administered. Half of the participants are told they are receiving active drug and half are told they are receiving placebo. In other words, they are apparently being unblinded at the start of the trial. In fact half of the participants are given an active drug and half are not, but with a crucial difference. Depending on assignment, participants will have one of four possible interventions: told they are getting the drug and do in fact receive it; told they are getting drug but in fact receive placebo; told they are getting placebo but in fact receive drug; and told they are getting placebo and in fact receive placebo. This permits independent and combined assessment of drug and placebo effects. Thus, half of the participants are misinformed about what they will receive and are debriefed *post-hoc*, after participation in the trial.

The balanced placebo design is useful because it provides a direct assessment of the drug effect, independent of expectancy. It has most commonly been deployed to test the role of expectancy on the effects of alcohol and caffeine consumption, usually with healthy volunteer samples [13,14]. It has the advantage that the told drug/get drug non-deceptive arm has more external valid than the double blind administration in conventional randomised trials, because they more accurately represent what happens in clinical practice. It has not yet been used in clinical trials of antidepressant medication, because of the ethical issues involved with temporary deception. However it is necessary to consider the merits of adopting an approach along these lines, if the question of the actual efficacy or otherwise of antidepressant medication is ever to be resolved.

### Ethics and pragmatics

The ethical problems with conducting a trial involving an element of deception are legion. Valid consent is established as a basic tenet of health care research, and a design involving temporary deception appears to fly in the face of this fundamental ethical principle. In order for consent to be valid, potential participants need to be able to make a decision in the light of all the relevant information about the proposed research procedure, including information about the nature, purpose, major risks and benefits of the procedure. Failure to obtain valid consent entails a lack of respect for the autonomy of persons, and is also likely to undermine patient's trust in health care professionals and medical research, a result which would clearly not be conducive to the long term interest of patients. Moreover, failure to be properly informed is likely to result in the *harm *of potential participants and others, either in terms of the distress that subsequent knowledge of deception may cause participants and others, and/or in terms of the significant risks to which deception may expose participants and others: for example if a participant decided to misuse a drug substance, believing it to be a placebo.

There are also many possible obstacles at the clinical level. Doctors might refuse to support such a trial or recruit patients for it. They might be convinced of the value of antidepressants, concerned by the amount of time it would take them to explain the trial's rationale to their patients, or worried by the prospect of creating such a high degree of uncertainty at a very delicate time in their patients' lives. They might also worry that this would have a damaging effect on the essential sense of trust that exists between themselves and their patients. The general public might consider all of the above reasons as valid, and wonder why there are still so many unanswered questions about a treatment approach that they had previously taken for granted.

Two approaches have been suggested to minimize such difficulties: pre-consent (including authorized deception and generic pre-consent) and minimised deception

#### Pre-consent

The principle of authorized deception has been advocated by Wendler and Miller [15]. They propose that subjects can be informed that a particular study involves deception, and asked to consent to its use, without being informed of the nature of the deception. Such a design has been used, for example, in the study of outreach stroke care [16].

This may offer a useful way forward. Although potential participants will be unaware of the nature of the deception, their autonomy would still seem to be respected because they will have consented to the deception Of course, participants would be very interested in trying to second guess which aspects of the study were being misdescribed, and this is likely to have unpredictable influences on the main question. A further issue is the extent to which the risks and benefits of a study can be determined without recourse to the potential participants' own views on the study – the point being that different aspects of a study may pose different ethical, religious or personal concerns for different participants. There may be no satisfactory way of checking this without first discussing the details of the study with potential participants. Finally, there is also the possibility that knowledge of deception taking place – even if consented to – might still cause participants some degree of anxiety, a possibility that ought to be avoided especially given the types of participants that would be recruited. However if the above concerns can be met, then authorized deception may represent a more acceptable study design to those participating in such a trial.

##### Generic pre-consent

Patients who are likely to be offered antidepressants – i.e. those who have had at least one previous treated episode of major depression – could be pre-consented to take part in a balanced placebo trial in the future. This might be ethically more acceptable to potential participants, but might affect the placebo arm by introducing a high (and unwanted) level of uncertainty in the minds of study participants. Moreover, consent is generally recognized as a process, not a one-off event. Potential participants may change their minds over the course of time for a variety of reasons, and therefore it cannot be assumed that an earlier act of consent will necessarily be valid at a later date.

#### Minimised deception

The degree of deception could be reduced by randomizing two-thirds/one third rather than half/half, and introducing the word 'probably'. Instead of patients being told "you will receive a placebo", they could be told "it is probable that you will receive a placebo", and the randomisation would then be two-thirds in favour of a placebo. This means that the information given to patients at the time they consented was correct, in that the majority of those told they would get a placebo, would in fact get a placebo. The statement that "it is probable that you will receive a placebo" is not by any means as definitive as "you will get a placebo" but it does not deceive the patient, since it is formally accurate.

### Study questions

To clarify these issues, prior to deciding whether a trial involving temporary deception could be feasible or ethically permissible, it would be helpful to gauge opinion amongst those groups of people who would be involved in such a trial: people with experience of depression, and (as most UK antidepressant trials recruit patients through them) general practitioners and psychiatrists. If it could be shown that sufficient of these groups were convinced of the validity and value of such a study, this might give added weight to the justification for such a trial.

We therefore sought to gather opinion from potential participants on two key questions:

1. Would a balanced placebo study of antidepressants be ethically acceptable to those who would take part in it?

2. Would a balanced placebo study of antidepressants be pragmatic, i.e. would potential participants be prepared to take part in such a study?

We also sought opinion as to whether it would make a difference to views on ethics and participation if subjects were pre-consented, or if deception were minimised.

## Methods

We sought opinions on the ethics and pragmatics of introducing an element of deception into antidepressant drug trials from members of the following groups of people living or working within the city of Liverpool:

1. General practitioners (GPs).

2. Psychiatrists.

3. People diagnosed with at least one episode of depression.

### Identification and recruitment

The names and contact details of all GPs and psychiatrists registered as working within Liverpool were identified through Liverpool Primary Care Trusts and Mersey Care NHS Trust. In order to minimize bias GPs and psychiatrists with a particular interest in the subject, or knowledge of members of the study team, were excluded from the study. 250 GPs were eligible for inclusion in the study. 125 of these GP's were randomly selected to be included in the study. The list of eligible GPs were arranged alphabetically and assigned numbers from 1 to 250. A physical random number generator (based on optical quantum physics) was employed. Duplications were permitted so that the probability of GPs being selected remained constant. All 65 eligible psychiatrists were selected for inclusion in the study.

Those selected were contacted by letter with an invitation to take part. These people were then contacted by telephone two weeks after receiving the letter, to discuss participation in the study. Those responding positively were given a participant information sheet and invited to complete the participant consent form. Those who agreed were asked to complete a brief questionnaire, indicating amongst other things, their length of time in practice, the extent of their involvement with mental health work, and their views on the efficacy of antidepressant medication. Purposive samples for interview were then created, based on the screening questionnaire responses. The main criterion for selection was to obtain a sample which reflected a range of opinion about antidepressant medication. We also took account of estimated proportion of patients' depressed [GP's only], special role in research, special role in mental health [GP's only], gender, number of GP partners [GP's only] and time since completion of training, when determining the precise composition of the interview sample.

People with at least one diagnosed episode of depression were identified through the practice records of those GPs who agreed to participate in the study. GPs wrote to suitable patients about the study and invited them to contact the research team. Those patients who did so were followed up by JGH. JGH arranged to meet them to discuss any concerns they might have, give them the participant information sheet and study consent form, and invite those who consented to the study to complete a brief questionnaire. This included questions on current mental state, opinion towards antidepressant medication, and age, gender and educational level.

### Data gathering

*Focus groups *were the data-gathering method of choice. This type of abstract and conceptual issue is often well suited to focus groups as the interaction helps people to explore and refine their own perspectives, attitudes and beliefs.

#### In-depth interviews

It was envisaged in designing the study that some participants might not be willing or able to join a group, or might feel uncomfortable about talking openly in a group. We therefore offered in-depth individual interviews as an alternative to participation in a focus group.

The focus groups and interviews sought the opinions of participants on the acceptability and feasibility of introducing an element of temporary deception into antidepressant drug trials. The participants were briefed in the invitation letter and in the introduction to the interview or focus group on the issues to be discussed (see Acknowledgements). A vignette-based topic guide invited participants' views on three different strategies for deception: post-hoc, authorised, and minimised deception (See Vignettes 1–3).

##### Vignette1: Post-hoc deception

*Peter is feeling miserable and out of sorts, and decides to go and see his family doctor. Peter and his doctor agree that he is suffering from depression, and that antidepressant medication might be a helpful treatment for him. Peter is then invited to take part in a trial, and is told that he will receive either an antidepressant or a placebo. He will be told which one he is taking. Peter agrees to take part. After the trial is over, Peter is told that he had not, after all, been fully informed about what happened. Half of the people who were told they were given the antidepressant medication, were in fact given the placebo. And half of the people who were told they were given the placebo, were in fact given the antidepressant*.

##### Vignette 2: Authorised deception

*First, imagine the same scenario as before. This time, however, you are told in advance that there will be an element of temporary deception in the trial. You are not told exactly what that element will be. You will be given a sheet of paper something like this: "You should be aware that the investigators have intentionally misdescribed certain aspects of this study. This use of deception is necessary to conduct the study. However, an independent ethics panel has determined that this form accurately describes the major risks and benefits of the study. The investigators will explain the misdescribed aspects of the study to you at the end of your participation"*.

##### Vignette 3: Minimised deception

*Imagine that you go to see your doctor when you are feeling depressed, as before. You agree to take part in an antidepressant drug trial. This time you are told either, 'you will probably receive 'placebo', or, you will probably receive an antidepressant'. You agree to take part*.

*After the trial is over, this is what you find out. One third of the people who were told they were given the antidepressant medication, were in fact given the placebo. And one third of the people who were told they were given the placebo, were in fact given the antidepressant. This is set out in *Table 1. *Since you had been told that you would probably get either antidepressant or placebo, there was not – technically – any deception involved in this trial*.

**Table 1:** to be inserted into vignette 3

	'You will probably get a placebo'	'You will probably get an antidepressant'
Given placebo	67%	33%
Given drug	33%	67%

Ethical approval for the study was granted by Liverpool Paediatric Research Ethics Committee (reference 04/Q1502/83).

### Analysis

All interviews and focus groups were audio-taped and transcribed verbatim. Transcripts were analysed thematically using *Framework*, a manual, matrix method, which facilitates thematic and cross-case interpretation [17,18]. Analysis proceeded in five stages:

• Familiarization. Transcripts were read and re-read by all the research team to familiarize and immerse in the data.

• Identification of the thematic framework. Meetings were held with the whole research team to discuss and identify Key issues, concepts and themes arising from the data, and to group them thematically to construct a conceptual framework.

• Indexing. Two of the research team (JGH and JJH) independently applied the thematic framework to the same transcript to explore any differences in application. The thematic framework was then applied systematically to all the data by JGH. The point of saturation of themes (i.e. no new themes emerging from new data coded) was reached before the end of the coding of the data set.

• Charting. JGH constructed thematic matrices for all identified categories/subcategories to further summarise and synthesise the indexed data. One of the transcripts was also independently charted by JJH to explore any differences in charting.

• Detection, categorization and classification. The original research questions were reconsidered by the research team, and the charts examined in order to define concepts, map the range and nature of phenomena, find any associations and provide explanations.

## Results

### Samples

Twenty seven general practitioners, 15 psychiatrists and six patients with experience of depression agreed to take part in the study and completed screening questionnaires.

Two focus groups and two in-depth interviews were conducted with a total of 14 GPs, of whom six were female. They reported a wide range of experience in general practice, and of expertise in mental health and research. Thirteen GPs considered antidepressants to be probably helpful in treating depression, the other was uncertain.

One focus group and five in-depth interviews were conducted with a total of eight psychiatrists, of whom four were female. Five were consultant psychiatrists, while three were specialist registrars. Two psychiatrists felt antidepressants were necessary in the treatment of depression, four that they were probably helpful, with two indicating they were unsure whether antidepressants were helpful.

Five interviews were conducted with patients, of whom three were female. Their age range was 37–50, and all but one had received more than two years education since age 16. All had experience of taking antidepressants, with three reporting themselves as currently depressed. Four patients perceived antidepressants as probably helpful, while one saw them as necessary in treating depression.

Further information on the numbers of participants taking part in focus groups or individual interviews is given in Table 2 and a summary of the key characteristics of the study sample is given in Table 3.

**Table 2:** Study sample by data gathering method

	GPs	Psychiatrists	Patients
Focus groups	12 (7,5)*	3	0
Interviews	2	5	5
TOTAL	14	8	5

* Brackets indicate: focus group 1 = 7 participants, focus group 2 = 5 participants

**Table 3:** Study sample (focus groups and interviews)

	GPs	Psychiatrists	Patients
Gender:			
Male	8	4	2
Female	6	4	3
Views on antidepressants			
Necessary	0	2	1
Probably helpful	13	4	4
Not sure	1	2	0
Probably unhelpful	0	0	0
Unhelpful	0	0	0
*Patients:*			
Currently depressed			3
Not currently depressed			2
Used anti-depressants			5
Never used a-d*			0
<2 yrs post-16 educ			4
>2 yrs post-16 educ			1
*Psyciatrists:*			
Consultant		5	
Registrar		3	
Consultant for <5 yrs		2	
Consultant 5–20 yrs		3	
Consultant >20 yrs		0	
*GPs:*			
Special role in mh**	4		
No special role in mh	10		
Special role in research	4		
No special role in res.	10		
Qualified for <5 yrs	0		
Qualified 5–20 yrs	6		
Qualified >20 yrs	8		
proportion of their patients perceived as being depressed:			
<10%	2		
10–30%	9		
>30%	3		

* anti-depressants, **mental health

### Participants' views on deception in clinical trials

Many participants accepted the need to consider alternative approaches to research into antidepressants. Based on participants' prior knowledge and experience, concerns were expressed at the lack of an evidence base to support the prescribing of antidepressant medication, particularly for mild or moderate depression. There was a desire for research to be conducted which more accurately reveals the pharmacological effect of antidepressants:

*"I mean is the medical professional deceiving itself at the moment in terms of its belief that antidepressants work?...Are we all being hoodwinked at the moment in terms of the fact that we believe that antidepressants work and they don't?" *GP 10

Participants generally did not view deception as being intrinsically unacceptable. They spoke about "acts of omission" and being "economical with the truth" in clinical practice, and the "little white lies" in everyday life. Only one, a psychiatrist, saw it as intrinsically unethical to introduce deception into any research setting. All participants could see the potential utility of introducing deception into trial designs.

However views on the ethical acceptability of deception within antidepressant drug trials varied significantly, and no consensus was found on any of the presented strategies.

*Post-hoc deception*, where participants are informed that the clinical trial involved an element of deception only after the completion of the trial, was considered unacceptable by the overwhelming majority of participants. Objections were predominantly based on concerns for the welfare of patients taking part in post-hoc deceptive trials, and a perceived moral and legal obligation for researchers to obtain valid informed consent.

"*I think as soon as you go into outright lies, as soon as that becomes the practice of the study you're crossing a line, and I think, I think ethically it's probably unacceptable." *Patient 02

*Authorised deception*, where participants give prior consent to take part in a trial which involves an element of deception, was the most commonly accepted strategy. However some participants argued that this strategy would reduce the pragmatic effectiveness of the study because participants may correctly deduce the element of deception: and hence that, if an authorised deceptive trial where unlikely to generate valid findings, it should not be conducted.

*"I think, you know, if you consent people to deception then ethically I haven't got a problem with it. But I can't see the point because you might as well just do a placebo controlled trial" *GP 08

*Minimised deception*, involving the use of ambiguous language to mislead participants on the probability of being assigned to treatment arms, was considered acceptable by many participants. However, with minimised deception the "devil's in the detail", with acceptability being inextricably linked to the precise wording of the information provided to potential participants, and the statistical probability of participants being assigned to the various treatment arms.

"The devil's in the detail and the wording is all important. And it has to convey a reasonably accurate analysis of the situation, whilst ... trying to eradicate the placebo effect as much as possible."

Patient 02

When asked whether they would likely be willing to participate in a deceptive trial of antidepressants opinion varied considerably. One patient, two GPs and three psychiatrists indicated that they would be unlikely to recruit for, or participate in, any of the presented strategies for deceptive trials. With one psychiatrist and two patients stating that they would be likely to participate in any of the deceptive trials presented, and were the only participants who indicated they would participate in a post hoc deceptive trial of antidepressants. For these three participants the main motivational factor was a desire to further knowledge of antidepressant efficacy, and the opportunity to contribute to improvements in the treatment of other patients with depression.

*"I think I'd do anything to help researchers really.... I would try all three. If I can't recruit I can't, but I wouldn't say no." *Psychiatrist 04

However the patients indicated that their participation would likely be dependent on their condition at that point in time, with them being more amenable to participation if their condition was currently impacting on their quality of life. and certain trial practicalities, such as the monitoring procedures. The hope of gaining alleviation from their depressive symptoms was also found to be a factor in their willingness to participate in any trial.

*"I think I'd take part in all of them..... I think from my point of view, if I felt I still had elements of a depressive illness and I was still being held back in a way, by the symptoms of depressive illness, I would take part in the survey, hypothetically as you say. If I felt that I'd reached a stage with my depression that it was no longer a factor in a) my working life, b) my social life, c) my domestic life, then I wouldn't, because you're on the straight and narrow and you don't want anything to demur from that or jeopardise it. But if, you know, I think you know with depression you have a feeling of dissatisfaction with the condition. And if the condition is still such that you're dissatisfied with it and you feel there are still elements of it that are affecting you, then yes, hypothetically*." Patient 02.

*"I think my reaction is such well because you know how horrible the situation can be, if you are doing something which can actually perhaps alleviate that horror, well then why not, its not going to harm me, so I think yes I probably would." *Patient 06

Of those who would agree to participate in a deceptive trial, the authorised deceptive design was the trial strategy which GP's and psychiatrists indicated they would be most willing to recruit for. In contrast the deceptive trial design which patients were most likely to agree to participate in was the minimised deceptive trial, with all five patients indicating that they would likely participate in such a trial.

### Factors influencing participants' views

The major issues that affected participants' views about the acceptability of deception studies were

• the welfare and capacity of patients

• practicalities of trial design

• the question of trust

• benefits.

#### Welfare and capacity

Participants from all of our samples (GPs, psychiatrists and patients) were concerned about the *welfare *of patients participating in deceptive trials. These included risks to patients from not receiving antidepressants, increased risks to patients if doctors were also deceived about their patient's treatment, and the need to ensure adequate monitoring processes and means of removing those patients whose condition deteriorates:

*"...if they were doing weekly Beck scores or something, I suppose that, and asked directly you know, how are you feeling, are you happy with your medication, and you know some check of their mood and their safety. Then maybe, yeah maybe this could be acceptable actually this deception." *Psychiatrist 07, discussing post-hoc deception.

The *capacity *of patients with depression, particularly severe or longstanding depression, to provide valid informed consent was a cause for concern. Participants considered that the complex nature of authorised or minimised deception would make it more difficult than usual to obtain valid informed consent:

*"I mean, the issue is if a person is really truly depressed, to what extent is he truly autonomous? To what extent is he or she in a position to make a decision, you know, in terms of giving their consent to a trial with all the informed information that goes with it?" *GP21

#### Practicalities

There was considerable discussion about *practicalities*, about the specific aspects of trial characteristics which might make any deceptive trial more, or less, acceptable. There was general agreement that, in addition to detailed consent procedures and clear methods for monitoring patients, acceptability would be enhanced if the trial were brief, and the post-trial disclosure of high quality:

*"I think the full disclosure of information at the end would go some way to persuade you in like your feelings about the wrongness of the deception. " *Patient 01

*"But a timescale would be important. So they could say, "yeah I can go with this for a couple of weeks but I wouldn't be able to go with it for two months," for instance. So the problem with the antidepressants is the kick in time varies but it's going to take quite a long time, so to me one of the relevant bits of this would be how long you would be deceiving them for." *GP 22

Some participants thought that the opinions and endorsement of valued individuals and organisations, including the responsible doctor, would help to reassure them about the acceptability of deception within trials:

*"I know things are hard to get through ethics committees and panels so it would, it would give me confidence in that way, that it has been like properly determined and everything, it's not just somebody coming up with the idea of I'm going to go out and do this. But its about hmm, see I would discuss it with my GP and see what they felt, and not saying I would be totally swayed by them but I would want, be interested in their opinion." *Patient 03

#### Trust

The question of *trust *was critical, and contentious, particularly with regard to the post-hoc deception scenarios. Many participants considered trust, whether in the research process or the doctor-patient relationship, to be of paramount importance.

*"I personally would be feeling really fucking pissed off if, to discover afterwards that I thought I was being told the truth. Because part of taking part in a trial involves a degree of trust in the people who are conducting it." *GP 09 (referring to reaction to participating in a post-hoc deceptive trial).

*"I don't think it can be justified by the greater good, you know because I think it's such a serious undermining of trust that there is no greater good in terms of slightly improved survey results or whatever, I think the greater good lies with the trust and not breaking that." *Patient 02 (discussing post-hoc deception).

*"The whole basis on which we operate is that people trust us to have their best intentions at heart and if we are part of deliberately deceiving them, where does that end. You can't say, I know I lied to you about this trial but I'll be honest about your mum or your kids or your own health. It doesn't work, once it's contaminated its contaminated." *GP 12

There were mixed views as to whether or not trust between doctor and patient would be adversely affected if the doctor, as well as the patient, were blinded to the deception. GPs and psychiatrists were more likely to prefer to be blinded, because they felt this would preserve their relationship with their patients and absolve themselves from blame in the patients' eyes.

*"I would feel more comfortable as a GP, with a GP hat on, if I didn't know that the patient *[was being deceived],*because I would feel that I'm more truthful, honest with the patient, rather than, what would I have to be, dishonest. Doctor patient relationship would be put at risk, I feel. I would be much happier if I wasn't informed." *GP 23

Patients, by contrast, were inclined to prefer their doctors to be 'in on' the deception, based on a belief in protective power of physicians' knowledge, and would tend to still hold the doctor culpable.

*"Well it's still coming from somewhere isn't it and well that shouldn't be allowed anyway, because it's just coming from, it's got to come from somewhere in the beginning. So we have put our trust in the GP, they should be knowing.....They should know. So they should be telling us." *Patient 04

There were also concerns about the consequences for trust in future research, if precedents for deception were to be set. Would studies like this be the beginning of a slippery slope?

*"The worry at another level it is sort of then where do we stop? I mean we have these ethical discussions about so many issues now, treatment and this, that, and all the rest of that, so where do we, where does the deception stop then? This study could lead to another study where it involved deception, and the deception could be at a higher level, and involve more and more risk. And then if you set a precedent and do this study, then of course people will go to that precedent and say that got approved and we could do this one as well." *PSY06

#### Benefits

A minority of participants thought that the balance of risks and benefits could be in favour of deception trials: that the greater good for patients in general from such studies could outweigh the potential for harm to an individual patient, or to their relationship with their doctor.

*"I wouldn't be cross at all if that happened, because I know, in a kind of the ins and outs of what happens in research and why it should happen. So if I've agreed then I wouldn't worry about anything else after." *Psychiatrist 04 (referring to perspective of a patient taking part in a post-hoc deceptive trial of antidepressants)

*"If we don't do research like this that involves doing something and deceiving the patient, there is a risk that you will harm other patients, and part of the ethical dilemma, I think, is in deciding whereabouts your tipping point is." *GP 14

## Discussion

This study is the first to ask those people who would be likely to be invited to take part in an antidepressant drug trial involving elements of deception, about their views of the ethical and practical issues involved in conducting such a trial. General practitioners, psychiatrists and patients with experience of depression are the three groups of people who most commonly take part in antidepressant drug trials. Their opinions are therefore critical in determining whether balanced placebo designs have a viable future in antidepressant drug trials.

Our techniques for recruitment and sampling were designed to generate as wide a range of opinion as possible. We noted that the majority of participants from all three groups considered antidepressant medication to be probably helpful, with only three participants expressing uncertainty about their efficacy. All patient participants had experience of antidepressant medication, though not all had found it personally effective. We think it probable that this range of views represents the diversity of opinion of both the professional groups involved in our study, and of those patients with experience of depression who decide to engage with primary healthcare. However biases are possible, with regard to the specificity of the locality and the likelihood that those who agreed to take part had a particular interest in the subject. We recruited fewer patients than anticipated, in part because of the constraints of having to go through the general practitioner. It is possible that those patients selected by their general practitioner were chosen on the basis of having a story to tell, or because their views were likely to be congruent with those of their doctor.

Many of the issues raised by the participants in this study apply to all randomised controlled trials, not just to those potentially involving deception. We accept, however, that the complexity of study designs involving balanced placebo options makes informed consent even more difficult to obtain than in conventional trials. We consider that this may pose particular problems with trials involving depressed people, given concerns about reduced mental capacity.

The underlying problem with authorised deception identified by our participants – that participants may correctly guess the deceptive element of the trial – has also been acknowledged by Miller et al [19]. To date this has only been formally tested in trials involving psychology students. It will be important to consider the extent to which this problem applies with other subjects and in other settings.

Alternative trial designs can be considered. One option, used successfully by Colloca and colleagues in an analgesia trial, is to adopt a combination of placebo run-ins and informed consent [20]. In this design all study participants are informed that they will start with a placebo. They are told that an active drug may be substituted after a while; and that they may (or may not) be informed when this switch is made. This protocol gives evidence for three of the four arms of a classic balanced placebo design – excluding 'told drug/no drug' – without any element of deception.

## Conclusion

There are legitimate concerns about the designs of current randomised placebo controlled trials of antidepressant medication, and the consequent limited validity of evidence arising from such trials.

Balanced placebo designs may be theoretically useful to address current design flaws. However, bringing deception into trials of antidepressant medication is both ethically and practically problematic. There is a trade-off between pragmatic and ethical responses to the question of whether, and under what circumstances, elements of deception could be introduced into antidepressant drug trials. Ensuring adequate ethical safeguards within balanced placebo designs is likely to diminish their ability to address the crucial issue of additivity. Participants in our study, who were deliberately recruited from those groups of doctors and patients likely to take part in any such trial in the future, could see substantial inherent problems with all proposed options for studies which included elements of deception.

Our conclusion, therefore, is that the balanced placebo designs considered in this study may not be feasible for introduction into future trials of antidepressant medication.

Given that the evidence-base for current antidepressant prescribing practices is equivocal, there remains an urgent need for improvement in trial design. This is necessary if we are to improve the quality of evidence about the utility and limitations of antidepressant medication, and the significance or otherwise of associated placebo characteristics, in order to help patients in distress and doctors in routine clinical practice. Alternative study designs, including placebo run-ins with informed consent, offer potential benefits. There may also be valuable lessons to be learned from a systematic re-examination of a range of placebo or contextual characteristics reported in previously published studies.

## Competing interests

The author(s) declare that they have no competing interests.

## Authors' contributions

CD conceived of the study, participated in its design, coordination and analysis, and drafted the manuscript. JGH participated in the coordination and fieldwork and undertook the primary qualitative analysis. JJH participated in the design, coordination, and fieldwork, and had oversight of the analysis. MW participated in the design, coordination, fieldwork and analysis. TW participated in the design, coordination and analysis of the study. All authors read and approved the final manuscript.

## Pre-publication history

The pre-publication history for this paper can be accessed here:


